# Microbial Contamination and Food Safety (Volume I)

**DOI:** 10.3390/biology14010059

**Published:** 2025-01-13

**Authors:** Joana Bastos Barbosa

**Affiliations:** Universidade Católica Portuguesa, CBQF—Centro de Biotecnologia e Química Fina—Laboratório Associado, Escola Superior de Biotecnologia, Rua Diego Botelho 1327, 4169-005 Porto, Portugal; jbarbosa@ucp.pt

## 1. Introduction

Food safety is a global concern driven by the constant need to find innovative solutions to mitigate contamination risks by pathogenic microorganisms. It is one of the main challenges of the food chain, from farm to fork/plate. According to the WHO [1], about 600 million people fall ill after consuming contaminated food, and 420 thousand die every year. Furthermore, the microbial contamination of foods causes huge economic impact due to productivity loss, product loss, recall costs, healthcare expenditure, increased insurance costs, legal expenses, and the loss of consumer confidence. Since microbial contamination can occur at any stage of the food chain, implementing effective food safety strategies is needed throughout production, postharvest handling, processing, distribution, and consumer handling to control and eliminate potential microbial hazards [2].

This Special Issue presents several topics focusing on the microbiological quality of different food sources, biofilm formation, food safety and quality monitoring practices, preservation technologies, and innovative approaches to enhance food safety and quality. After the peer review process, twenty-two original research articles were included in this Special Issue of *Biology*.

## 2. Special Issue Overview

Cleaning procedures prevent contamination and ensure food safety, but its constant validation is essential in the agri-food industry. After conducting a survey in 37 French agri-food industries, the first of its kind in France, De Oliveira Mota et al. [3], from the French Agency for Food, Environmental and Occupational Health & Safety and SECALIM, INRAE (France), highlighted the importance of environmental monitoring practices (EMPs)—which are site-dependent and aim to consider the product, process, production volume, and location—in ensuring food safety and identified a need for the guidance and harmonisation of practices. Still, on the topic of the systematic monitoring of microbial contamination, Lupattelli et al. [4], from the University of Perugia and Istituto Zooprofilattico Sperimentale dell’Umbria e delle Marche “Togo Rosati” (Italy), presented a five-year study assessing the microbial quality and safety of meals and work surfaces in collective catering systems (schools and hospitals) in central Italy. With the results obtained, the authors emphasised the need for continuous monitoring in collective catering systems to ensure food safety, particularly for vulnerable populations.

Focusing on microbial contamination and its implications, several authors have examined different food sources, geographical locations, and specific microorganisms. Beindorf et al. [5], from the Veterinary Faculty, Institute for Infectious Diseases and Zoonosis, Centre for Clinical Veterinary Medicine (Germany) and University of Veterinary Medicine Vienna (Austria), examined the microbiological quality and safety of ostrich meat samples from farms in Bavaria, Germany, and studied the antimicrobial resistance of strains from ostrich meat and stool samples. The authors found two meat samples positive for STEC genes as responsible for producing Shiga toxins and 13 isolated *Enterobacteriaceae* as resistant to antimicrobials considered critically important by the WHO. These results establish baseline data for future hygiene control procedures and monitoring programs for ostrich meat production. Chien et al. [6], from the National Kaohsiung University of Science and Technology, National Taiwan Ocean University, and Kaohsiung Medical University (Taiwan), analysed the microbial and chemical quality of mustard pickle products. No foodborne pathogens were found in the samples, although the sulphite content was over 100 ppm, well above the 30 ppm limit set by food additive legislation in Taiwan. This demonstrates the need for strict control of food additives and microbial contamination in fermented products. El-Hossary et al. [7], from Zagazig University (Egypt), Taif University, and King Abdulaziz University (Saudi Arabia), investigated *Aeromonas* spp. in raw fish markets and humans in Egypt, considering antibiotic resistance, virulence, and biofilm formation. High levels of antibiotic resistance were detected, and biofilm formation capacity was observed in some isolates, highlighting the potential for the transmission of multidrug-resistant *Aeromonas* spp. through the food chain, posing a significant public health risk. In addition, Ballah et al. [8], from Bangladesh Agricultural University (Bangladesh), investigated the presence of biofilm-forming *Staphylococcus aureus* in various food samples and human hand swabs in Bangladesh. A significant number of isolates, detected in 23.81% of the samples, were found to be biofilm producers. They used both qualitative and quantitative tests, highlighting the potential public health risk associated with contaminated food sources and poor hygiene practices. Regarding pathogens, such as *S. aureus*, that also colonise skin and soft tissues, Lizardo et al. [9], from CBQF, Universidade Católica Portuguesa (Portugal), showed promising results with the adhesion of probiotic bacteria to skin keratinocytes and the underlying mechanisms aimed at inhibiting the adhesion of pathogens to keratinocytes. Among other results, emphasis should be given to the role of probiotic *Lacticaseibacillus rhamnosus*, which demonstrated a wound healing capacity and reduced *S. aureus* counts in ex vivo models, suggesting its potential use in treating skin infections.

The concept of biofilm formation emerges as a significant factor contributing to the persistence of certain pathogens on various surfaces, further challenging food safety management. Salamandane et al. [10], from the University of Lisboa (Portugal), Lúrio University (Mozambique), and the National Centre for Scientific Research (Angola), found multidrug resistance in 68.8% of enterococci isolated from cheese, and 100.0% of isolates exhibited a biofilm-forming ability. The authors also found that although the isolates were sensitive to benzalkonium chloride (MIC ≤ 10 mg/L), the biofilms of the same isolates became resistant to higher concentrations (80 mg/mL) tested. Effective countermeasures are vital and different materials and substances with antimicrobial properties are constantly being explored to combat microbial contamination and enhance food safety. Khayat et al. [11], from King Abdulaziz University, Prince Sattam Bin Abdulaziz University, Imam Mohammad Ibn Saud Islamic University (Saudi Arabia), Zagazig University, Suez Canal University, Ain Shams University (Egypt), Virginia Commonwealth University (USA), and Oman College of Health Sciences (Oman), showed that sodium citrate significantly reduced biofilm formation by more than 60% and other virulence factors of *Serratia marcescens*, such as motility and the production of prodigiosin, protease, and hemolysins. Xylia et al. [12], from the Cyprus University of Technology (Cyprus), reported the potential of Cypriot oregano (*Origanum dubium*) essential oil and hydrosol in reducing *Salmonella enterica* and *Listeria monocytogenes* on tomatoes and cucumbers, with greater efficacy against *L. monocytogenes*, even seven days after application and storage at 11 °C. Also, regarding the contamination of fresh produce, Truschi et al. [13], from the University of Florence and Institute of Applied Physics ‘Nello Carrara’ (Italy), identified that leaf roughness and water content are positively correlated with *Escherichia coli* attachment on baby leafy greens, suggesting that these characteristics can increase the risk of contamination. Moreover, the authors also reported that leaf roughness can affect the effectiveness of UV treatment in reducing bacterial contamination.

Studies that delve into the effectiveness of surface coatings and natural compounds against pathogens address the pressing concerns of bacterial resistance and cross-contamination in food-related environments. Dias et al. [14], from the Faculty of Technology Cariri, Regional University of Cariri and Nucleus of Technology and Industrial Quality of Ceará (Brazil), Research Center for Biosciences and Health Technologies (Portugal), and the University of Las Palmas de Gran Canaria (Spain), reported the antibacterial potential and synergistic effect of fixed oil from *Artocarpus heterophyllus* almond against standard and multidrug-resistant bacterial strains. Di Cerbo et al. [15], from the Universities of Camerino, Bergamo and Modena and Reggio Emilia, CNR-Nanoscience Institute-S3 and Moma Nanotech S.r.l. (Italy), and National Agricultural Research Centre (Pakistan), investigated the bactericidal effect of a special anodising method, DURALTI^®^, based on TiO_2_ nanoparticles deposited on aluminium discs and subjected to UV and 70% alcohol and the time necessary to achieve this effect against several pathogenic bacteria. The authors confirmed the ability of the DURALTI^®^ coating to effectively reduce 4 logs of all bacteria tested after 6 h and a general inhibition within 15 s of UV exposure and up to 1 min after alcohol disinfection. Bento de Carvalho et al. [16], from CBQF, Universidade Católica Portuguesa (Portugal), assessed the effectiveness and durability of a quaternary ammonium compound-based surface coating in reducing surface contamination. The coating demonstrated efficacy against all pathogens tested, with a reduction greater than 5.0 log CFU/cm^2^ in less than 1 min on all surfaces, and it maintained its antimicrobial efficacy even after the surfaces were cleaned with bleach, a damp cloth, a commercial degreaser, and a commercial disinfectant.

Three studies explored the valuable tool of challenge testing in evaluating the efficacy of interventions and processes in controlling pathogens, providing information on approaches such as carbonation [17], phage therapy [18], and fermentation with protective cultures [19]. Schalli et al. [17], from the Medical University of Graz (Austria), investigated the effect of three different concentrations of carbon dioxide (CO_2_) on the survival of *S. aureus* and *Enterococcus faecalis*, artificially added to bottled mineral water. The study suggests that although carbonation alone does not replace the need for proper disinfection and cleaning procedures in bottling lines, its use can be considered an additional measure to ensure water safety, especially in regions with microbial contamination problems. Cui et al. [18], from Shandong Agricultural University and Poultry Research Institute of Shandong Academy of Agricultural Sciences (China), assessed the effectiveness of phage CKT1 therapy in controlling the vertical transmission of *Salmonella* Pullorum in artificially infected adult broiler breeders. Phage CKT1 therapy effectively reduced the pathogen colonisation in various tissues and reproductive organs and reduced the risk of vertical transmission, suggesting that phage therapy may be a viable alternative to antibiotics in controlling *Salmonella* infections in poultry. Martín et al. [19], from the University of Extremadura (Spain), evaluated the effect of dry-cured fermented “salchichón” processing, using a selected strain of *Lactilactobacillus sakei*, on the growth and virulence gene expression of *L. monocytogenes*. The processing of “salchichón” reduced the counts of *L. monocytogenes*, and adding *L. sakei* resulted in an additional decrease. Additionally, the combined effect of reduced bacterial counts and decreased virulence gene expression indicates that the “salchichón” processing effectively controls the hazard posed by this pathogen.

Particularly effective for fresh, minimally processed items such as juices, meat, seafood, and dairy products, the non-thermal preservation technology of high-pressure processing (HPP) meets the growing demand for safe and high-quality clean-label foods [20]. Huang et al. [21], from the National Kaohsiung University of Science and Technology, Yuanpei University of Medical Technology, and Kaohsiung Medical University (Taiwan), explored the effect of combining brine salting (3 and 9% NaCl) with HPP (300, 400, 500, and 600 MPa pressure for 5 min) on mackerel fillets and concluded that combining a 3% brine solution with HPP at 400 MPa improved the quality and safety of mackerel fillets. Komora et al. [22], from CBQF, Universidade Católica Portuguesa, National Institute of Health Dr. Ricardo Jorge, and University of Aveiro (Portugal), assessed the impact of HPP (300 MPa, 5 min, 10 °C) combined with biocontrol (bacteriophage Listex and bacteriocin-producing *Pediococcus acidilactici*) on the bacterial communities and quality of a fermented meat sausage model. The authors found that HPP-assisted biocontrol had minimal impact on the bacterial communities’ structure and composition of fermented sausage and did not significantly alter its quality parameters (colour, texture, and lipid peroxidation) during refrigerated storage. Huang et al. [23], from the National Kaohsiung University of Science and Technology and Kaohsiung Medical University (Taiwan), investigated the impact of HPP (250, 350, 450, and 550 MPa for 5 min) on controlling histamine-forming bacteria (*Morganella morganii* and *Photobacterium phosphoreum*) in tuna meat. The authors suggested that HPP treatment at a minimum of 350 MPa for 5 min can effectively inhibit the growth of histamine-forming bacteria and reduce the risk of histamine poisoning in tuna meat. Hwang et al. [24], from the National Kaohsiung University of Science and Technology, Yuanpei University of Medical Technology, and Kaohsiung Medical University (Taiwan), examined the inactivation of foodborne pathogens in carrot juice using HPP (200–600 MPa, 0.1–15 min) and its impact during refrigerated storage. The authors concluded that although effective in inhibiting and delaying the growth of *Salmonella* Typhimurium (300 MPa, 3 min) and *E. coli* (400 MPa, 3 min), HPP was not effective at inhibiting the growth of *L. monocytogenes* in carrot juice during cold storage, which highlights the risk of foodborne illness in refrigerated fruit juices treated by HPP.

A new method for bacterial colony counting (a time-consuming method) using few-shot learning and an edge computing device was introduced by Zhang et al. [25], from The Hong Kong University of Science and Technology and Clearwaterbay Biomaterials Ltd. (China). The proposed method uses the Random Cover Targets Algorithm to augment data and train a model with only five original images, and it can run on low-cost devices with a high accuracy (97.4%) and detection speed and a false negative rate of only 1.5%. This offers a practical solution for water and food quality control applications for on-site testing with minimal data requirements.

In summary, the different papers published in this Special Issue highlight the need for continuous surveillance and intervention strategies since food safety is a significant global concern. I want to thank the authors for their valuable and instructive contributions to this Special Issue. I would also like to acknowledge all the reviewers for their precious help and the staff at the editorial office of *Biology*, with a special thanks to the Section Managing Editor, May Tang, for their incredible support.
